# Improved 3D-ResNet sign language recognition algorithm with enhanced hand features

**DOI:** 10.1038/s41598-022-21636-z

**Published:** 2022-10-24

**Authors:** Shiqi Wang, Kankan Wang, Tingping Yang, Yiming Li, Di Fan

**Affiliations:** College of Electronic and Information Engineering, Shandong University of Science and Technology, Qingdao, 266590 Shandong China

**Keywords:** Engineering, Mathematics and computing

## Abstract

In sign language video, the hand region is small, the resolution is low, the motion speed is fast, and there are cross occlusion and blur phenomena, which have a great impact on sign language recognition rate and speed, and are important factors restricting sign language recognition performance. To solve these problems, this paper proposes an improved 3D-ResNet sign language recognition algorithm with enhanced hand features, aiming to highlight the features of both hands, solve the problem of missing more effective information when relying only on global features, and improve the accuracy of sign language recognition. The proposed method has two improvements. Firstly, the algorithm detects the left and right hand regions based on the improved EfficientDet network, uses the improved Bi-FPN module and dual channel and spatial attention module are used to enhance the detection ability of the network for small targets like hand. Secondly, the improved residual module is used to improve the 3D-ResNet18 network to extract sign language features. The global, the left-hand and the right-hand image sequences are divided into three branches for feature extraction and fusion, so as to strengthen the attention to hand features, strengthen the representation ability of sign language features, and achieve the purpose of improving the accuracy of sign language recognition. In order to verify the performance of this algorithm, a series of experiments are carried out on CSL dataset. For example, in the experiments of hand detection algorithm and sign language recognition algorithm, the performance indicators such as Top-N, mAP, FLOPs and Parm are applied to find the optimal algorithm framework. The experimental results show that the Top1 recognition accuracy of this algorithm reaches 91.12%, which is more than 10% higher than that of C3D, P3D and 3D-ResNet basic networks. From the performance indicators of Top-N, mAP, FLOPs, Parm and so on, the performance of the algorithm in this paper is better than several algorithms in recent three years, such as I3D+BLSTM, B3D ResNet, AM-ResC3D+RCNN and so on. The results show that the hand detection network with enhanced hand features and three-dimensional convolutional neural network proposed in this paper can achieve higher accuracy of sign language recognition.

## Introduction

Sign language is an important way of communication besides language. It expresses semantics through a series of hand and arm movements, supplemented by facial expressions, eyes, lips and so on. It is one of the effective ways for deaf-mutes to communicate with the outside world^1^. Sign language recognition can also be used for human–computer interaction. It has broad prospects in intelligent driving, multimedia teaching, smart home, medical treatment, virtual reality, industrial application and other fields^2,3^.

The related research of sign language recognition has been widely concerned. According to different data acquisition methods, it can be divided into the recognition algorithm based on wearable devices and the recognition algorithm based on vision. The recognition algorithm based on wearable devices^4–6^ collects the data information of hand movement in real time through sensors, and then recognizes it through a series of algorithms. Such algorithms have high recognition accuracy, but most wearable devices (such as data gloves, EMG sensors and so on.) are expensive and need to be carried around, which is difficult to popularize in daily life. The recognition algorithm based on vision collects images or videos through the camera, and uses image processing or deep learning algorithm to realize sign language recognition without wearing equipment. For example, Hidden Markov Model (HMM)^7,8^, Conditional Random Field (CRF)^9^, Dynamic Time Warping (DTW)^10^ and so on, which recognize sign language through artificially designed features. Most of the features extracted by such algorithms are shallow feature information, which is difficult to have good robustness in practical application scenarios.

The method based on deep learning can extract better semantic features of sign language and obtain better recognition effect, which is a hot research topic of video sign language recognition at present. Escobedo et al.^11^ mapped the position and movement information of the hand to the texture feature map, and then used multi-channel CNN network to recognize the sign language video and the result was that the fused features have better classification and representation ability. Borg et al.^12^ proposed multi-layer RNN for sign language recognition. The inputs of the network are RGB image and optical flow image. First, CNN is used to extract the features of each frame of an image, and then RNN is used to time sequence model the features. The classification performance of the model has been significantly improved. An et al.^13^ used ResNet34 network to extract spatial features and fused attention mechanism in LSTM to automatically learn the importance of each frame and improve the role of useful frames. Huang et al.^14^ introduced the spatial attention mechanism on the basis of 3D-CNN, focusing on regions of interest, and achieved an accuracy of 88.7% on the Chinese sign language dataset and 95.3% on the Chalearn14 dataset. Jiang et al.^15^ proposed a new framework for skeleton aware multimodal sign language recognition, proposed SSTCN (Separable Spatial Temporal Convolution Network) to extract skeleton features, used 3D-CNN to extract spatio-temporal information in RGB video and depth video, and finally integrated multimodal information.

In the application scenario of sign language recognition, hand detection is a key step in the whole algorithm process, which is used for subsequent recognition tasks. Through the analysis of the characteristics of hand motion, it can be seen that the hand area is small, the hand movement speed is fast, there are cross occlusion and other problems, therefore it is difficult to fully extract the hand motion information only through the global image recognition. The traditional hand detection algorithms^16–18^ mainly use the prior knowledge of hand shape, skin color, texture and so on, and extract features manually to complete the detection work. This kind of detection algorithm is relatively simple in calculation, less in computation, and does not need large-scale datasets, but its robustness is poor, and it is vulnerable to the influence of background, illumination, skin color and other factors.

In recent years, many target detection networks based on deep learning have emerged, such as Faster R-CNN^19^, YOLO-v3^20^, CenterNet^21^, EfficientDet^22^, which can be well applied to the field of sign language recognition. The related technology of target tracking^23–26^ is also a technical reference source for sign language recognition. In order to better detect small hand targets, Si et al.^27^ proposed R-FCN (Region-based Fully Convolutional Networks), designed a feature pyramid structure to facilitate the acquisition of detailed and highly semantic features, and established a large dataset from real classroom videos, with an average detection accuracy of 90%. Gao et al.^28^ improved the SSD network and proposed the FF-SSD (Feature-map Fused SSD) network to fuse the features of different convolution layers, effectively solving the problem of small hand size in the image. Xie et al.^29^ proposed a context attention feature pyramid network for human hand detection. The introduced CAM (Context Attention Module) captures context information while retaining local edge features, and achieves a detection accuracy of 97.5% on Oxford and VIVA datasets. It is also noted that several excellent algorithms in recent years are worth learning from. For example, Adaloglou et al.^30^ proposed an improved I3D framework and processed spatiotemporal features through BLSTM to model the correlation of long-term time, and used the dynamic pseudo label decoding method to iteratively train the entire architecture. Liao et al.^31^ proposed a multi-modal dynamic sign language recognition method based on deep Three-dimensional residual convolution network and bidirectional LSTM network, and named it BLSTM-3D residual network (B3DResNet). This model can solve complex gesture classification tasks and distinguish small differences from similar gestures between different people. Zhang et al.^32^ proposed a sign language recognition framework based on AM-ResC3D global feature analysis and Mask RCNN local feature description. Fakhfakh et al.^33^ proposed an algorithm to fuse dynamic and static features. The static level is the key point of the head/hand, and the dynamic level is the accumulation of the key point trajectory matrix. Xiao et al.^34^ proposed a skeleton based on the CSL recognition and generation framework based on recurrent neural network (RNN), this framework supports bidirectional CSL communication.

Although significant progress has been made in a sign language automatic recognition, it still faces many difficulties and challenges. For example, in sign language video, the hand area is small, the movement is complex, easy to cross block and blur, and the information is too much and difficult to capture. In addition, the dataset is small, the number of model parameters is large, the amount of feature extraction in time series is large, and the speed of model recognition is slow. This paper mainly studies the problem of isolated word sign language recognition, and proposes an improved 3D-ResNet sign language recognition algorithm to enhance hand features. The improved EfficientDet network is used to detect the hand region in the image, and the image sequences of left-hand and right-hand regions are input into the recognition network. The improved 3D-ResNet is used to extract the spatio-temporal features in the input sequence. Through the fusion and joint action of the three parts of features, the recognition rate of sign language is improved. How to accurately get the left and right hand regions and integrate their local and global features into the whole sign language recognition algorithm is the key problem to be solved in this study. In this paper, the CSL dataset is used to verify the proposed algorithm. Compared with other algorithms, it improves the accuracy of recognition results, improves the computing speed of convolution, and can extract more robust features.

The main contributions and innovations of this paper are as follows:

1. Aiming at the problem that the hand region is small in the video, which affects the accuracy of sign language recognition, an algorithm idea and framework of sign language recognition based on the fusion of two hand features and global features are proposed. From the experimental results, this idea and practice can effectively improve the extraction ability of key features and the recognition rate of sign language. This part is mainly stated in “Overall framework of the algorithm” section.
2. An improved hand detection model of EfficientDet network is studied and proposed, which strengthens feature extraction, integrates more levels of information in different scale feature layers, and improves the representation ability of features and the perceived ability of small hand targets. At the same time, the attention module of DCSAM is designed and applied to the improved enhanced feature extraction module, which enhances the ability of the model to extract spatial features, and the accuracy of hand detection is improved to a certain extent. This part is mainly stated in “Improved EfficientDet hand detection algorithm” section.
3. Research and improve the 3D-ResNet18 sign language recognition network. First, the network integrates the feature information of the hand region, and the global and local fusion features have better representation ability. Second, an improved residual module is designed to decompose the traditional three-dimensional convolution into two-dimensional convolution in space and one-dimensional convolution in time. By decomposing the traditional three-dimensional convolution, the parameter amount of the model is reduced. At the same time, the ME motion excitation module and the HSB module are integrated to enhance the extraction ability of the network for the motion information and spatial information in the input features. The model improves the accuracy of sign language recognition while reducing the parameters. This part is mainly stated in “Three features extraction and fusion based on improved 3D-ResNet18” section.

## Overall framework of the algorithm and hand detection algorithm

### Overall framework of the algorithm

**Figure 1:**
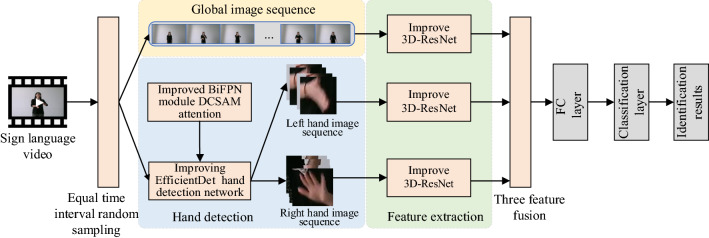
Framework of improved 3D-ResNet sign language recognition algorithm to enhance hand features.

The improved 3D-ResNet sign language recognition algorithm framework to enhance hand features is shown in Fig. 1. The algorithm is mainly divided into four parts: video frame sampling, hand detection, feature extraction and three features fusion. Video frame sampling divides the input sign language video into 16 video segments, and randomly selects a frame from each video segment to reduce redundant frames in the video. The hand detection network is based on and improved on EfficientDet and improves the Bi-FPN module. Through the process of up-sampling and down-sampling, the network can integrate features from different levels to solve the problem of poor perception of deep features to small targets. At the same time, the attention module of DCSAM is designed to strengthen the attention of the network to the useful information in the channel and space, and improve the accuracy of hand detection. After obtaining the global image and the left-hand and the right-hand image sequences, the improved 3D-ResNet18 network is used to extract the features respectively. In this paper, an improved residual module is designed, which decomposes the traditional three-dimensional convolution into two-dimensional convolution in space and one-dimensional convolution in time to reduce the amount of convolution calculation. At the same time, the motion excitation module is used to enhance the perception of motion information in the input sequence, and more refined features are extracted in the form of feature grouping to enhance the ability of feature representation. The extracted local features of the left and right hands are directly added, and then vertically spliced with the global features to obtain the classification features after the fusion of the three features. Finally, the sign language recognition results are obtained through the full connection layer and softmax classification layer.

### Improved EfficientDet hand detection algorithm

**Figure 2:**
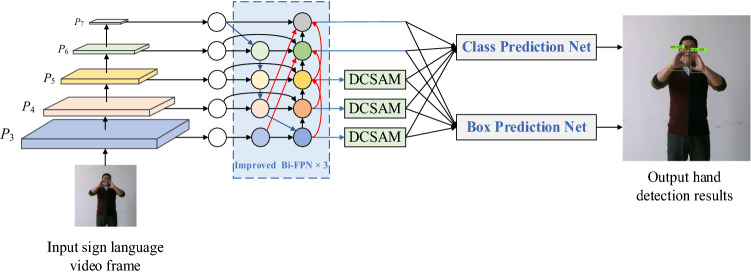
Framework of hand detection algorithm based on EfficientDet and integrating DCSAM attention.

**Figure 3:**
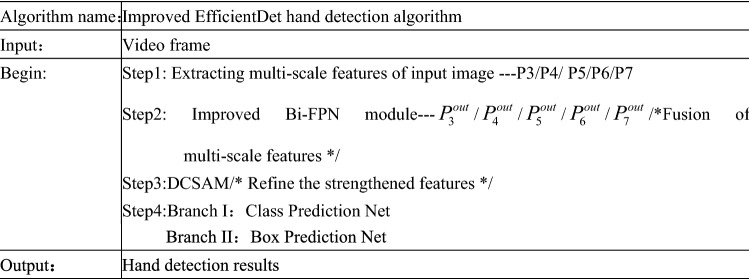
Pseudocode of improved EfficientDet hand detection algorithm.

Sign language mainly determines the meaning of expression through hand movements. If only the global image is used for sign language recognition, the robustness of the algorithm may be poor, and the recognition accuracy of more complex actions may be low. This paper constructs a hand detection algorithm based on EfficientDet and integrates DCSAM attention. Its framework is shown in Fig. 2. Firstly, multi-scale features of the input image are extracted; P3–P7 respectively represent feature maps of different scales, where P3 represents the largest feature map and P7 represents the smallest feature map. Then, the extracted features are up-sampled and down-sampled by the improved Bi-FPN feature enhancement module to realize multi-scale feature fusion. After that, the enhanced features are refined by using the designed dual channel and spatial attention module DCSAM, so that the network can pay more attention to useful information. Finally, the hand detection results are obtained through classification and frame regression network. The pseudocode of the improved EfficientDet hand detection algorithm is shown in Fig. 3.

**Figure 4:**
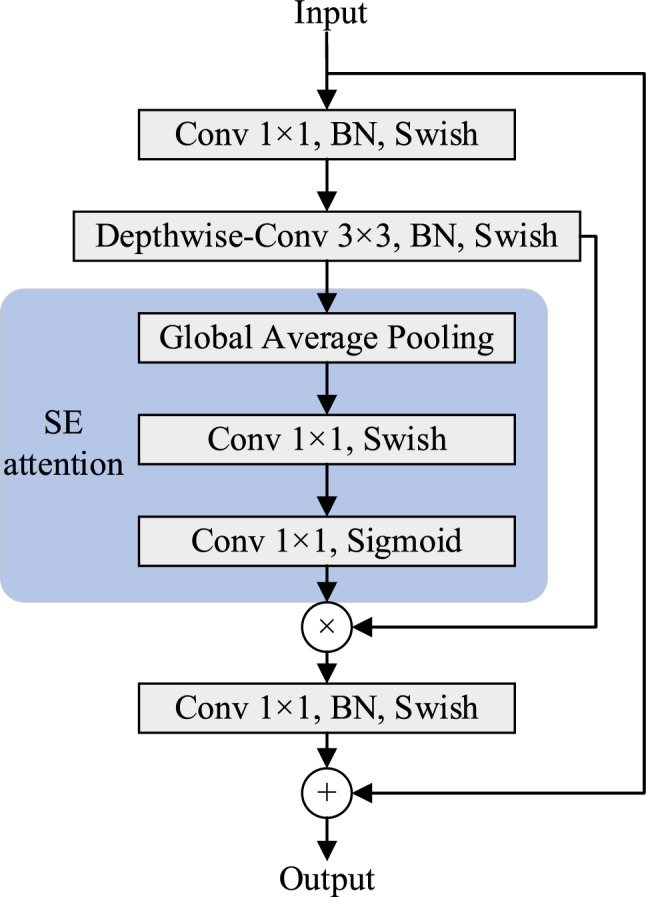
Convolution structure diagram of EfficientNet.

EfficientDet network^22^ is designed by the Google brain team. It has eight different scales D0–D7, corresponding to B0–B7 in EfficientNet feature extraction network^35^. By setting different parameters, the input image resolution, network depth and width can be adjusted. By weighing the relationship between the three, the model is more efficient and the detection accuracy is higher. For sign language recognition task, EfficientDet-D0 can meet the need of hand detection. It uses EfficientNet-B0 as the backbone feature extraction network, the size of the input image is 512 × 512, and three Bi-FPN modules are used to enhance feature extraction. EfficientNet network is stacked by multiple Mobile inverted Bottleneck Convolution (MBConv) modules, and SE attention module is applied. Figure 4 is the convolution structure diagram of EfficientNet^35^. In MBConv, in 1 × 1 convolution after adjusting the number of channels, the depth separable convolution is used to replace the traditional convolution, which reduces the parameters in the calculation process and makes the model lighter while maintaining accuracy. At the same time, after the deep separable convolution, the attention mechanism is introduced to make the network pay attention to the important parts. In addition, the network also uses the Swish activation function instead of the original Relu activation function to improve the overall performance of the network.

### Improved enhanced feature extraction module

**Figure 5:**
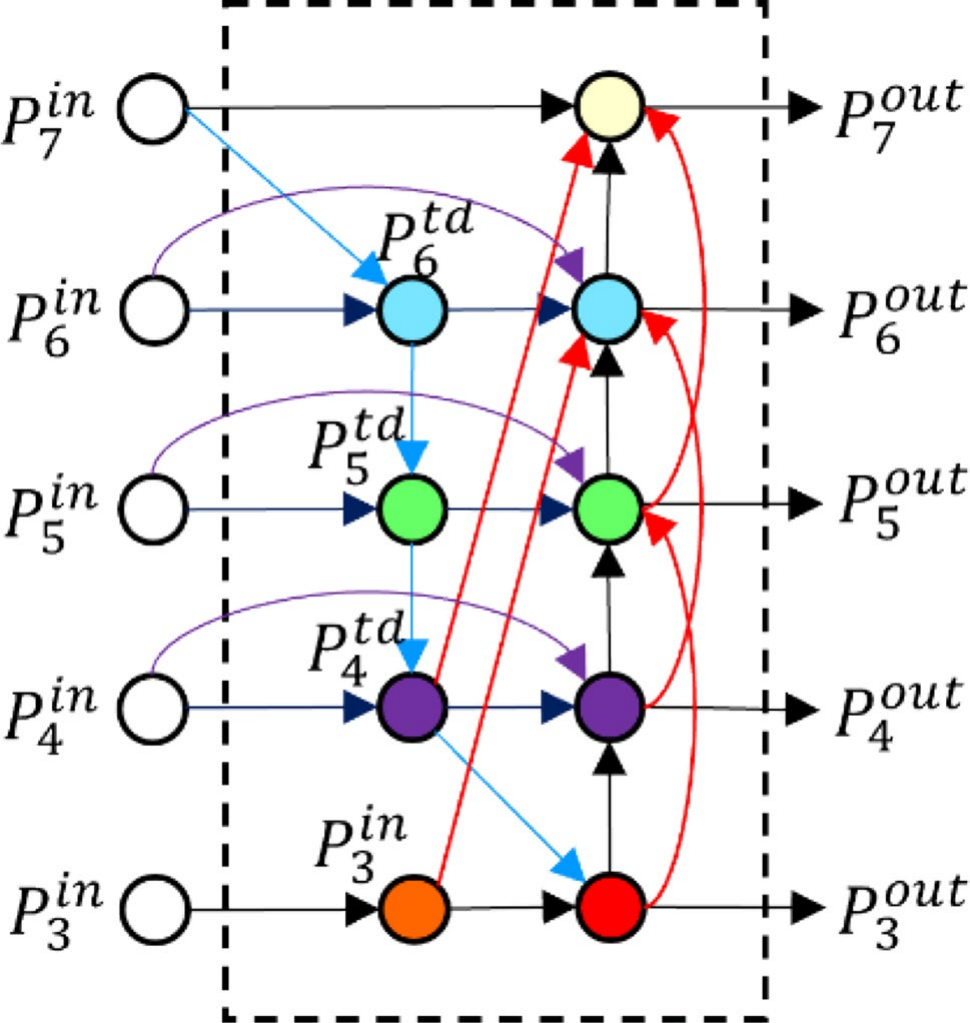
Bi-FPN module improved in this paper.

Multi-scale network design can extract more robust and high semantic information. It can not only extract the shallow features of the input image, but also extract its deep features. Therefore, in the detection task, multi-scale can better detect the target objects of different sizes. However, the size of features obtained from multiple feature layers will be different, so the process of up sampling and down sampling will be used in feature fusion. In this way, the final features include both the location information of shallow features and the high semantic information of deep features. Hand detection requires more refined features, not only accurate hand position information, but also rich high-level semantic information. In this paper, the Bi-FPN module^22^ is improved by adding cross-level connections on the basis of the original network to enhance the full use of features, as shown in Fig. 5.

**Figure 6:**
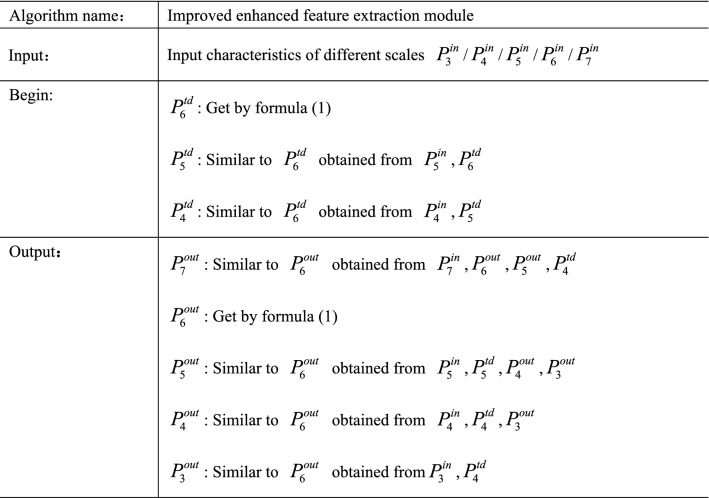
Improved enhanced feature extraction module pseudocode.

For input characteristics of different scales $P^{in} = [P_{3}^{in} ,P_{4}^{in} ,P_{5}^{in} ,P_{6}^{in} ,P_{7}^{in} ]$ with different levels of features, the corresponding output layer features are $P^{out} = [P_{3}^{out} ,P_{4}^{out} ,P_{5}^{out} ,P_{6}^{out} ,P_{7}^{out} ]$, and the intermediate sampling layer features are $P^{td} = [P_{4}^{td} ,P_{5}^{td} ,P_{6}^{td} ]$.Taking the layer $P_{6}$ as an example, the calculation method is:

1 $$\left\{ {\begin{array}{*{20}l} {P_{6}^{td} = conv\left( {\frac{{w_{1} \times P_{6}^{in} + w_{2} \times resize(P_{7}^{in} )}}{{w_{1} + w_{2} }}} \right)} \hfill \\ {P_{6}^{out} = conv\left( {P_{6}^{in} + P_{6}^{td} + resize\left( {P_{5}^{out} } \right) + resize\left( {P_{4}^{out} } \right) + resize\left( {P_{3}^{in} } \right)} \right)} \hfill \\ \end{array} } \right.$$

where $conv$ represents convolution operation, $resize$ represents up sampling or down sampling operation, $w_{1}$ and $w_{2}$ represents the weights of different scale features respectively. The pseudocode of the improved enhanced feature extraction module is shown in Fig. 6.

### Dual channel and spatial attention module

**Figure 7:**
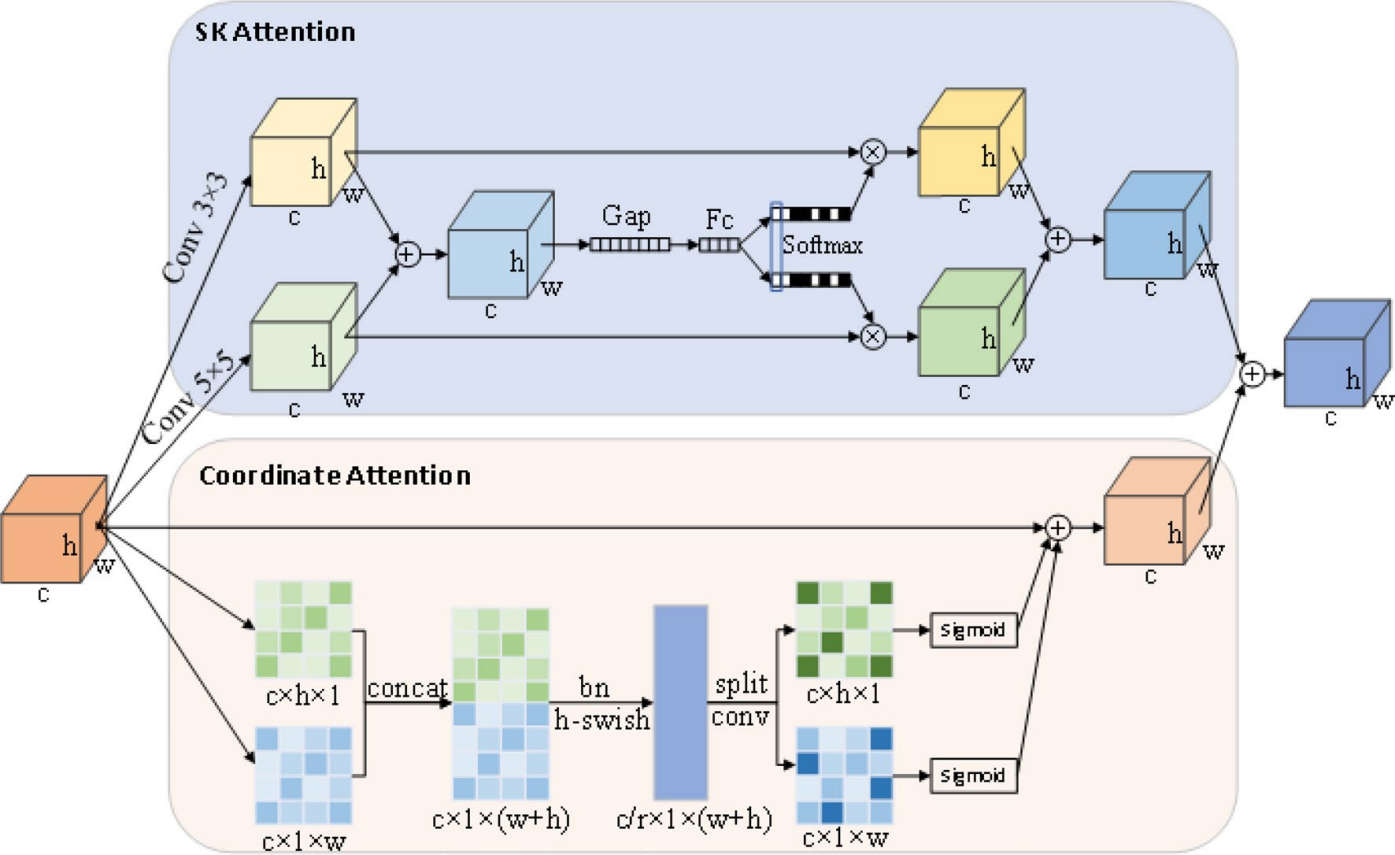
Designed DCSAM attention module.

The DCSAM attention module designed in this paper is shown in Fig. 7. Using the idea of parallel connection of channel attention and spatial position attention, SK attention^36^ and Coordinate Attention^37^ are fused.

**Figure 8:**
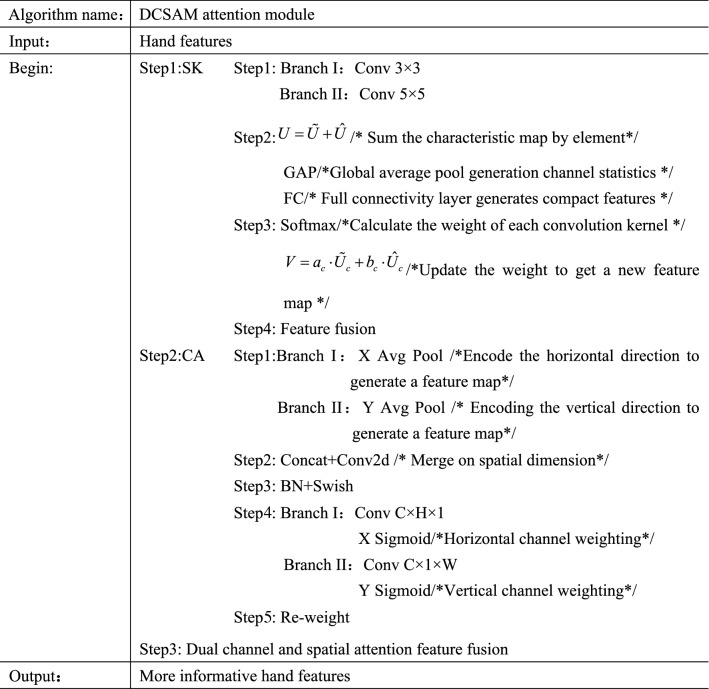
DCSAM attention module pseudocode.

Attention mechanism is very effective to improving the performance of the model, and can focus on useful information in a large amount of information. In the process of hand detection, the image of the hand region also has different sizes. In order to extract more abundant hand features, it is necessary not only to use channel attention to focus on important channels, but also to enhance the perception of hand position in space. In this paper, the DCSAM attention module is placed after the improvement of the Bi-FPN module. In view of the small size of the $P_{6}$ and $P_{7}$ layers, it is also found that the effect of adding attention after it is not ideal after the experiment, so it is only added after the $P_{3}$, $P_{4}$ and $P_{5}$ layers. Through the fusion of dual channel and spatial attention, the information in the features is more abundant; the network pays more attention to the features of interest, and effectively improves the accuracy of hand detection. The pseudocode of DCSAM attention module is shown in Fig. 8.

## Three features extraction and fusion based on improved 3D-ResNet18

### Improved 3D-ResNet18 network

Most 3D-CNN networks are directly improved on the basis of 2D-CNN network architecture. 3D convolution kernel is used to replace the original 2D convolution kernel, which is widely used in human behavior recognition, video understanding, analysis and other fields. However, sign language recognition is more complex and sophisticated than other recognition tasks. If 3D-CNN network is directly applied, it is difficult to obtain features containing rich spatio-temporal information, which will affect the speed and accuracy of sign language recognition.

**Figure 9:**
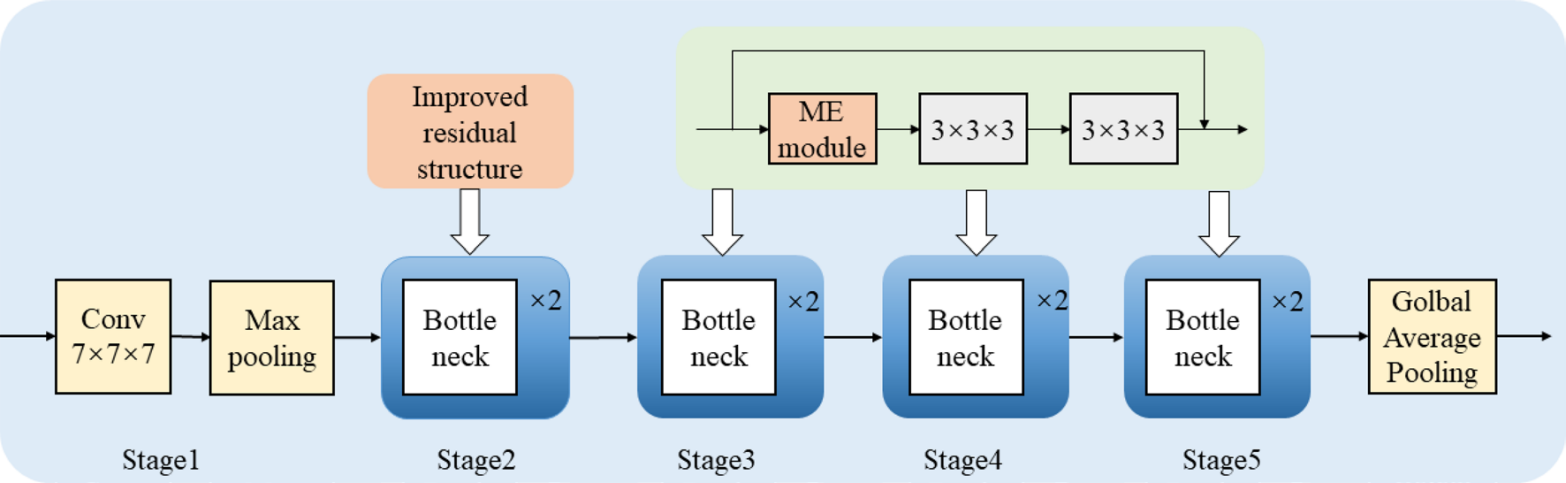
Improved 3D-ResNet18 network structure.

**Figure 10:**
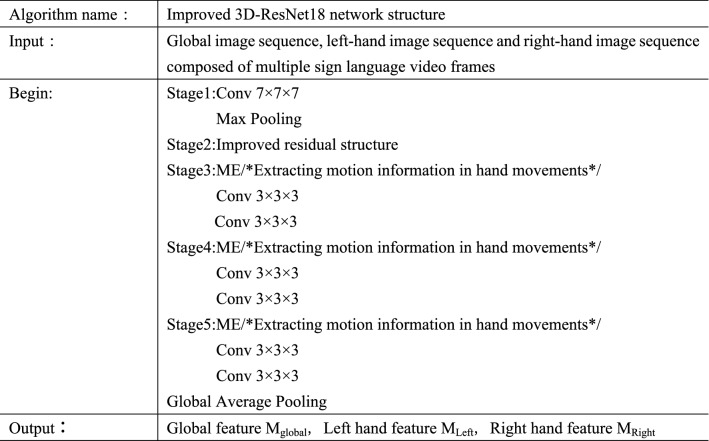
Improved 3D-ResNet18 network algorithm pseudocode.

Therefore, this paper improves the 3D-ResNet18^38^. The residual design enables the network to learn even when the network depth is deepened. The deeper network can extract higher-level features, so as to obtain effective sign language representation and improve the recognition accuracy. The improved residual module uses the idea of P3D network^39^ for reference, decomposes the three-dimensional convolution to reduce the computational cost, and integrates Motion Excitation (ME) module^40^ and Hierarchical-Split Block (HSB) module^41^, which can not only focus on the motion information in the input sequence, but also extract more refined features in the spatial domain. The 3D-ResNet18 network is stacked by multiple residual structures, as shown in Fig. 9. Its convolution layer can be divided into five stages. We put the improved residual module in Stage2, and Stage3, Stage4 and Stage5 only introduce ME module on the basis of the original residual module. The input image passes through the convolution layer, the maximum pooling layer and the global average pooling layer to obtain the classification features. The use of the maximum pooling layer effectively improves the calculation speed, reduces the influence of useless information and improves the robustness of the extracted features. The global average pooling layer aggregates the spatial information in the feature without parameter optimization, which effectively avoids the phenomenon of overfitting. The pseudocode of the improved 3D-ResNet18 algorithm is shown in Fig. 10.

**Figure 11:**
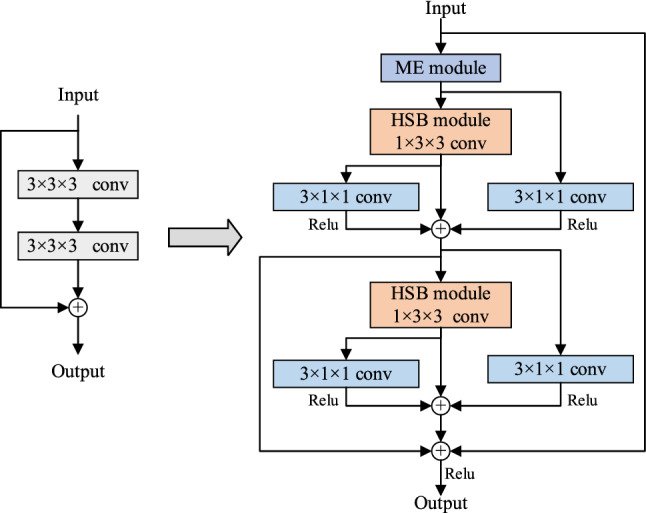
Improved residual structure.

**Figure 12:**
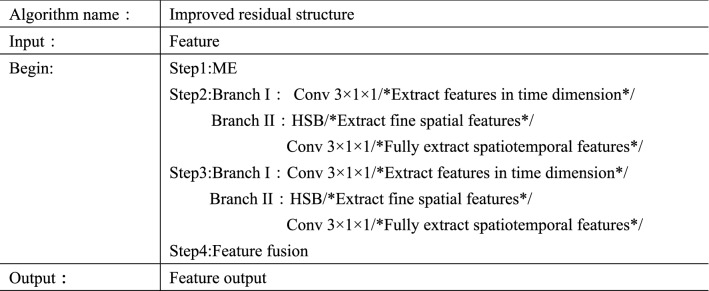
Pseudocode of improved residual network.

In the framework of sign language recognition in this paper, the input is an image sequence composed of multiple sign language video frames. In addition to feature extraction in the spatial domain of the image, it is also necessary to model the motion information of the action. Due to the characteristics of three-dimensional convolution itself, the direct use of three-dimensional convolution will increase the cost of calculation, and the decomposition of three-dimensional convolution into two-dimensional space convolution and one-dimensional time convolution will cause the loss of motion information. In order to better model the motion information in sign language and extract more accurate features, this paper improves the ME module^40^ and HSB module^41^ and designs a new residual structure, as shown in Fig. 11. In the new residual structure, the input feature first passes through the ME module to stimulate the sensitive motion channel in the feature, so that the feature fuses the motion weight at each time, and can better extract the motion information in the hand movement. Then the feature containing motion information is sent to two branches, part of which uses the convolution of 3 × 1 × 1 to extract the features in the time dimension. In the other part, HSB structure is used to extract the fine spatial features firstly, and then the convolution of 3 × 1 × 1 to fully extract its spatio-temporal features. Finally, the three features are fused with the input features to avoid the phenomenon of gradient disappearance or gradient explosion in the training process. The pseudocode of the improved residual network is shown in Fig. 12.

### Fusion method of three features

**Figure 13:**
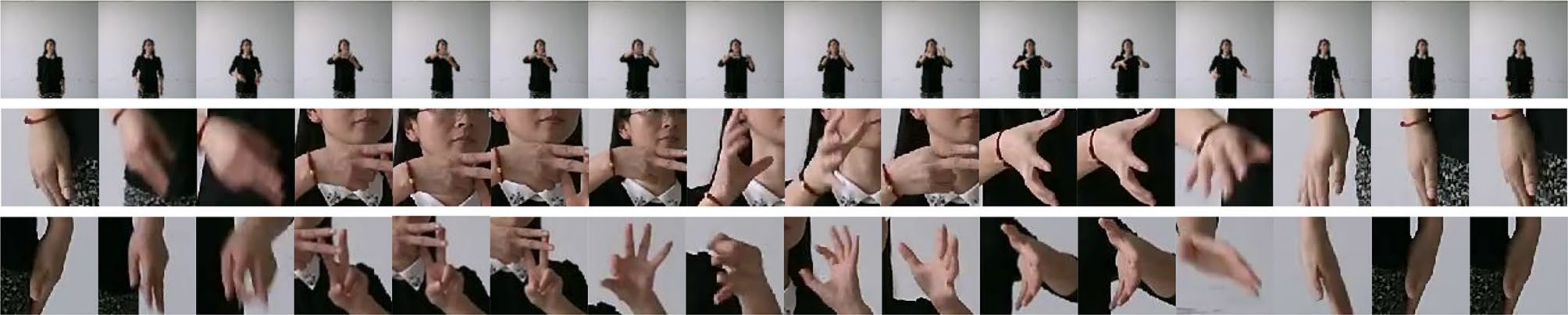
Left-hand and right-hand region image sequences.

At present, most of the sign language recognition methods are carried out on the global image, but ignore the attention to the details of the hand. In this paper, the global image sequence and the local area image sequences of the left and right hand are used as the input of the 3D-ResNet18 network to extract the global and local space–time features. The feature fusion of the hand region plays a very important role in improving the accuracy of the sign language recognition algorithm. The feature representation ability of the fused hand region is stronger, which helps to improve the performance of the model. Figure 13 is a left-hand and right-hand region image sequence extracted using the improved EfficientDet model.

The 3D-ResNet18 recognition network is divided into three parts. One branch uses the global image sequence as the input to extract the motion trajectory information in the input, and the other two branches are the left-hand and right-hand image sequences to focus on the shape and motion changes of the hand. The three branches use the same 3D-ResNet18 network structure, and their weights can be shared, the features after the Stage5 layer are selected for fusion, so as to converge the global and local features, which are represented by $M_{global}$, $M_{Left}$ and $M_{Right}$, respectively. First, use the average pooling operation $G$ to adjust the feature dimensions to obtain the global, left-handed and right-handed local features $C_{global}$, $C_{Left}$ and $C_{Right}$ respectively. The adjusted feature vector dimension is 512 × 1. The calculation is shown in formula (2).

2 $$C_{global} = G\left( {M_{global} } \right),C_{Left} = G\left( {M_{Left} } \right),C_{Right} = G\left( {M_{Right} } \right),$$

**Figure 14:**
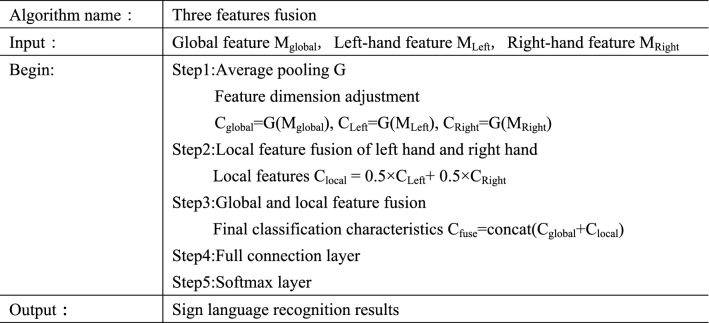
Pseudocode of three features fusion algorithm.

In the fusion stage, the algorithm uses the splicing operation to fuse the global and local features to obtain the final classification feature $C_{fuse}$, and the calculation is shown in formula (4).

3 $$C_{local} = 0.5 \times C_{Left} + 0.5 \times C_{Right}$$

4 $$C_{fuse} = concat\left( {C_{global} + C_{local} } \right)$$

where $C_{local}$ represents the local features including left-hand and right-hand features, $concat$ represents the splicing operation, and the final fused classification feature $C_{fuse}$ is a 1024 dimensional feature vector. After that, sign language recognition results are obtained through the full connection layer and softmax layer. The pseudocode of this algorithm is shown in Fig. 14.

## Experimental results and analysis

### Experimental environment and data

The sign language recognition algorithm experiment proposed in this paper is based on Pytorch deep learning framework and Python language programming, and uses a GPU graphics card to accelerate calculation. Pytorch is a commonly used deep learning framework at present. It encapsulates many functions for easy use, and the calculation method is dynamic graph calculation. It can flexibly adjust the structure of the network, and the operation speed is fast, efficient and concise. The specific environment configuration is shown in Table 1.

**Table 1:** Experimental environment configuration.

Configuration name	Parameter
Operating system	Windows 10
Graphics card	GeForce RTX 2070 (8G)
CPU	Intel(R) Core(TM) i7-10875H CPU @ 2.30 GHz
CUDA	CUDA 10.2 + cudnn7.6.5
Deep learning framework	PyTorch 1.5.0
Programing language	Python 3.7.7

**Figure 15:**
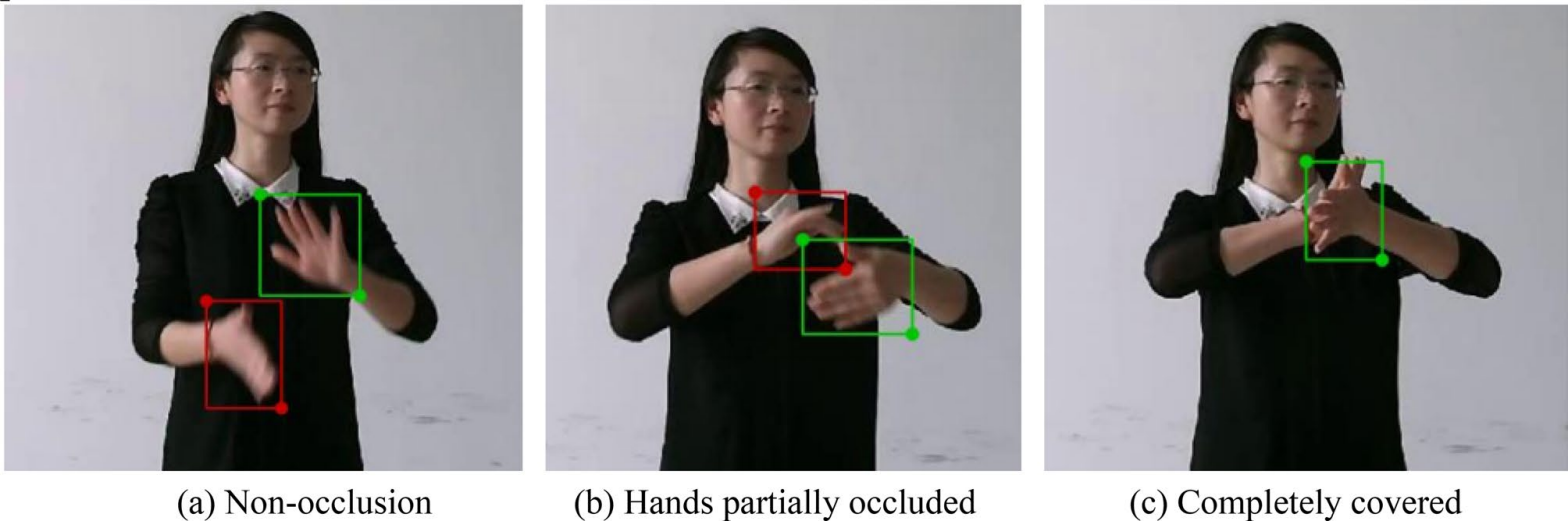
Schematic diagram of annotation image.

The dataset used in the experiment comes from the Chinese Sign Language (CSL) dataset constructed by Huang et al.^14^ of the University of science and technology of China. The dataset is taken under the Kinect camera. It has a large amount of data, diverse actions and data types, covers common vocabulary in life, and provides RGB video, depth video and skeleton point data. It contains 500 types of isolated word sign language videos, each type of action contains 250 video samples, which are made by 50 participants repeatedly shooting for 5 times. Hand detection requires a dataset with hand position annotation. However, most open source data sets only annotate the position of the hand, but do not distinguish between the left-hand and the right-hand. In order to verify the effectiveness of the algorithm in this paper, this paper constructs a hand detection data set based on the CSL dataset, selects 40 types of sign language action videos and extracts nearly 30,000 images from them, and uses Labelme software to label the left and right hand in the images. A total of 26,270 pieces of manually labeled data are included. The dataset is divided into a training set, test set and verification set according to 8:1:1, including 21,016 pieces of training set, 2627 pieces of test set and verification set respectively. The marking process is shown in Fig. 15. The marking content is divided into left hand and right hand. Figure 15a shows the case without occlusion. The rectangular boxes containing left and right hands are marked with the wrist as the boundary; Fig. 15b shows the marking under partial occlusion; In Fig. 15c, the left hand is completely covered, so only the visible part is marked.

### Experimental results and analysis of improved EfficientDet hand detection algorithm

In this section, the hand detection model based on EfficientDet and integrating DCSAM attention is tested. The dataset used is the hand detection dataset including own-labeled data and the data re-constructed from CSL dataset^14^. In the experiment, the selected detection network is EfficientDet-D0, the corresponding feature extraction network is EfficientNet-B0, the batchsize is 8, and the learning rate is 1 × 10^–4^, the number of training rounds is set to 100, and the Adamw optimizer is used for gradient descent. In this paper, the commonly used evaluation indexes of mAP (mean Average Precision), FLOPs (floating point operations), Parm (parameter), FPS (frame per second) and P-R (precision recall) curve are used to analyze and evaluate the experimental results. mAP is used to evaluate the effect of target detection and classification. The higher the mAP value, the better the algorithm performance. FLOPs are floating-point operands, which are used to measure the complexity and computation of the model. The larger the FLOPs, the more complex the calculation of the model and the greater the amount of calculation. Parm is the total number of parameters in the model, which is used to measure the complexity of the model. The larger Parm, the more complex the model. On the P-R curve, the vertical axis is the precision and the horizontal axis is the recall. When the area under the P-R curve is larger, the performance is better. FPS is the number of frames of detected images per second. The higher the frame rate, the faster the processing speed in the actual detection task.

**Figure 16:**
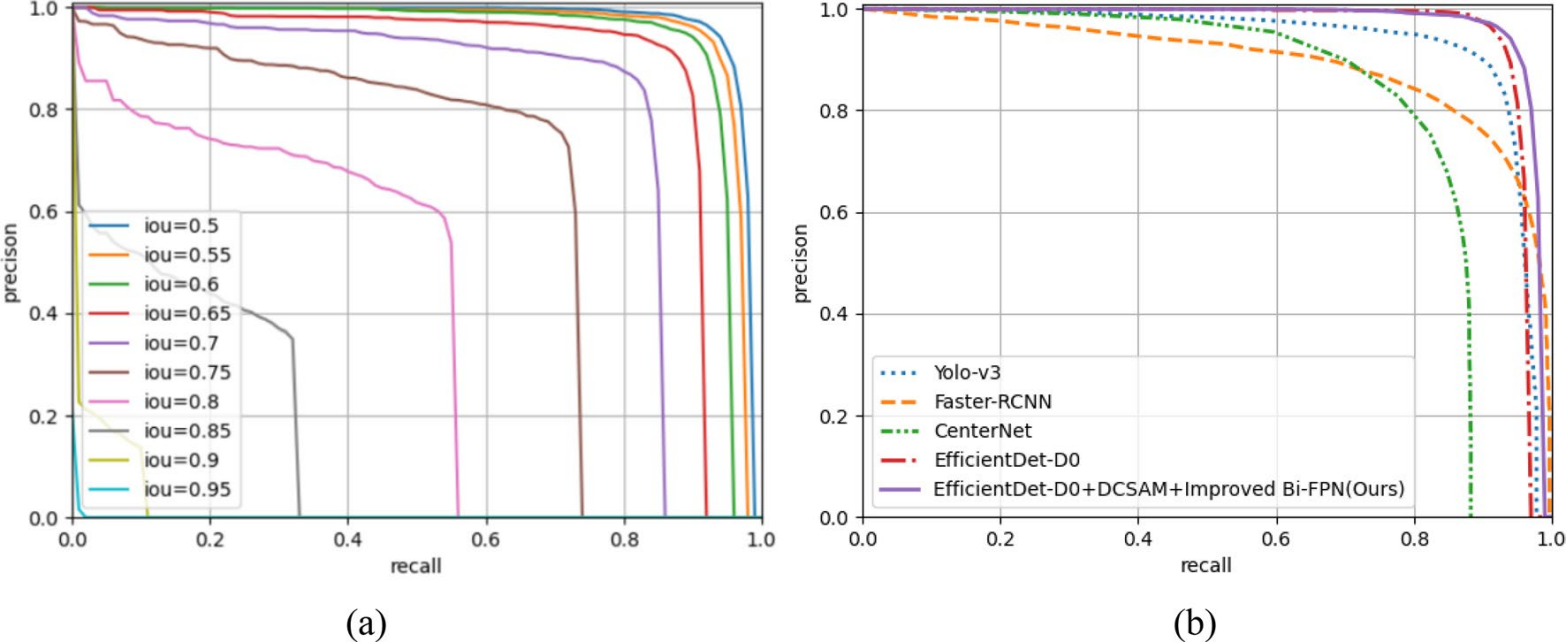
P-R curve comparison diagram, (**a**) P-R curve of this algorithm under different IOU thresholds, (**b**) P-R curve of different detection algorithms when IOU = 0.5.

We compared the P-R curve of the Faster-RCNN^21^, CenterNet^25^, Yolo-v3^24^ and the algorithm in this paper, which can compare the detection effect of the model intuitively and comprehensively. Figure 16a shows the P-R curve of the algorithm under different IOU thresholds. It can be seen that the area under the P-R curve is the largest when IOU = 0.5. Figure 16b shows the P-R curves of different detection algorithms when IOU = 0.5. The area under the P-R curve of the algorithm in this paper is the largest, which can explain that the detection performance of the model in this paper is relatively good to a certain extent.

In addition, this paper makes a comparative experiment with Faster-RCNN^19^, CenterNet^21^, Yolo-v3^20^ detection algorithms, and evaluates them from the four indicators of mAP, FLOPs, Parm and FPS. In the experiment, the feature extraction network used by Faster-RCNN and CenterNet is Resnet50, the backbone feature extraction network used by Yolo-v3 is Darknet53, and EfficientDet-D0 uses EfficientNet-B0 for multi-scale feature extraction. The experimental results are shown in Table 2.

**Table 2:** Comparison of mAP, FLOPs, Parm and FPS of different detection algorithm models.

Model	mAP (%)	FLOPs (G)	Parm (M)	FPS
Faster-RCNN	89.58	461.65	28.29	9.67
CenterNet	83.20	35.03	32.66	65.79
Yolo-v3	93.58	32.76	61.53	44.86
EfficientDet D0	94.24	2.28	3.83	26.41
EfficientDet D0 + SK	95.27	2.31	3.84	21.98
EfficientDet D0 + CA	95.66	2.30	3.84	20.91
EfficientDet D0 + DCSAM	96.11	2.34	3.84	20.19
**EfficientDet D0 + DCSAM + Improved Bi-FPN(Ours)**	**96.25**	**2.34**	**3.84**	**19.43**

Experimental results of final model are in bold.

As can be seen from Table 2, the Faster-RCNN algorithm has a large amount of floating-point operations and parameters, and the frame rate per second is only 9.67, which is difficult to meet the requirements of real-time detection. When CenterNet uses Resnet50 as the backbone network, its detection speed is relatively fast, but the detection accuracy is only 83.20%, which is lower than other algorithms. Yolo-v3 can have high detection accuracy, reaching 93.58%, but compared with the model in this paper, its floating-point operations and parameters are relatively large. The average detection accuracy of EfficientDet-D0 is 94.24%, and it also has obvious advantages in floating-point operation and parameter quantity, which are 2.28 and 3.83 respectively. In order to verify the effectiveness of the improved hand detection model, a comparative experiment is carried out for each module. After introducing the SK attention module and the coordinate attention module respectively, the average detection accuracy reaches more than 95%. After the introduction of the DCSAM designed in this paper, the map value of the model reaches 96.25%, which is a certain improvement compared with the EfficientDet-D0 model, and does not introduce too much computation, and the amount of floating-point operations and parameters does not increase much. The detection frame rate of the model reaches 19.43, which can meet the needs of hand detection.

**Figure 17:**
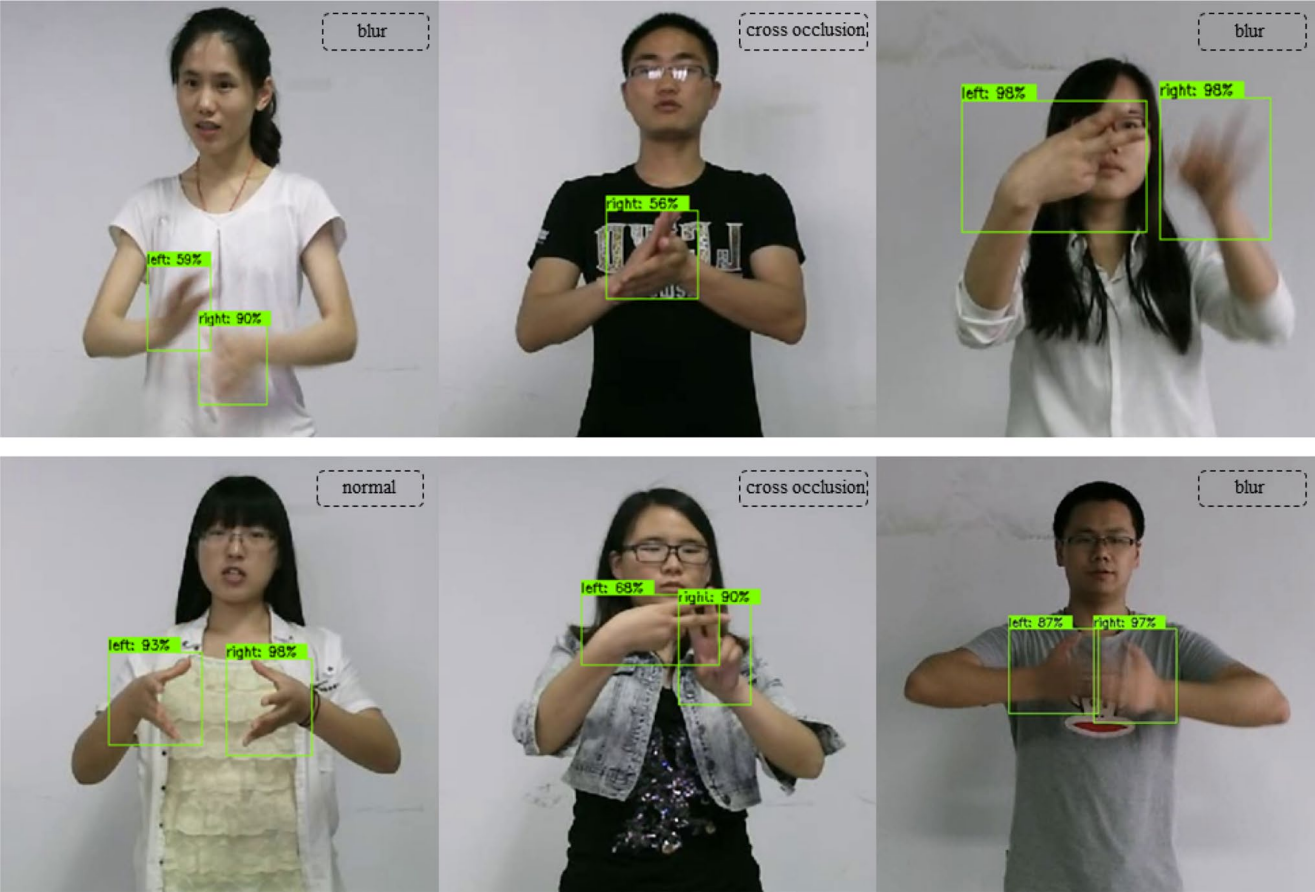
The hand detection effect of this model.

At the same time, in order to test the hand detection effect of this model, this paper selects images under several typical scenarios in the CSL dataset^14^ for verification. The detection effect is shown in Fig. 17. It can be seen from this that the hand area can be accurately detected in normal, cross occlusion and blur situations.

However, in practical application, there are many samples that are difficult to distinguish. In order to objectively determine the detection effect of the model in this paper, several images and the comparison algorithm are selected for testing, as shown in Table 3. It can be seen that the hand detection model based on EfficientDet and integrating DCSAM's attention proposed in this section effectively improves the detection accuracy of the model while ensuring the floating-point operation and parameter quantity. Through the improved Bi-FPN module, the feature extraction is strengthened, and the DCSAM attention is used to strengthen the focus on the small target area of the hand, so as to enhance the performance of the model.

**Table 3:**
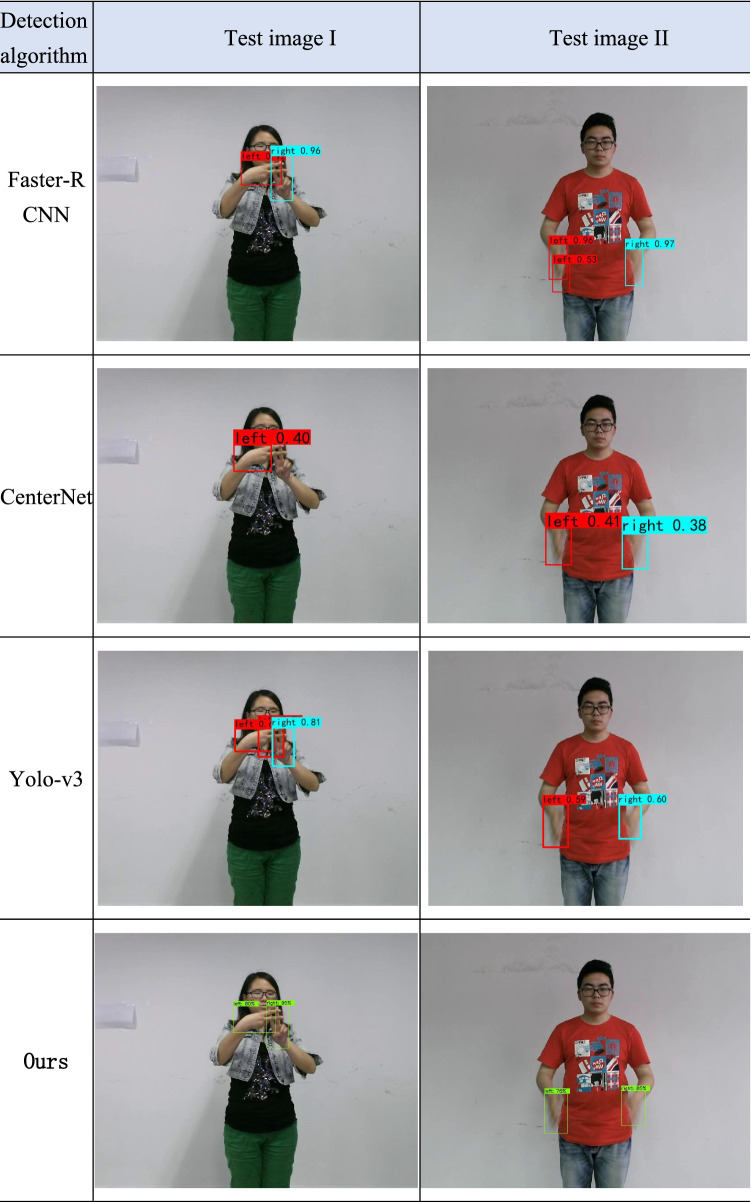
Comparison of detection effects of different algorithms.

### Experimental results and analysis of improved 3D-ResNet18 sign language recognition algorithm

We used the first 100 categories of isolated word sign language in the CSL dataset for the experiment. See “Experimental environment and data” section for details of the dataset. In order to reflect the recognition ability of the network for non-specific people, this paper divides the dataset into training set and test set, with a ratio of 8:2. The sign language actions of the first 40 people in each category are taken as the training set, and the sign language actions of the last 10 people are taken as the test set, which can ensure that the actors in the test have not appeared in the training process. Each sign language video extracts 16 video frames to represent the continuous action of sign language, and uses center clipping to adjust the size of the input image to 112 × 112 to eliminate the redundant background area, so that the input of the network is adjusted to 3 × 16 × 112 × 112. In the training process, the batch is set to 8, the number of rounds is 100, the initial learning rate is 0.03, and it decreases to 0.1 times when training 15, 30, 45, 60 rounds. The SGD optimizer is used for gradient descent. The weight attenuation coefficient is set to 0.005 and the momentum parameter is set to 0.9. The Loss value of training is calculated by the cross entropy loss function to measure the difference between the two probability distributions of network output and target value. At the same time, in order to avoid the overfitting of the network, this paper uses the data enhancement method to expand the data and improve the generalization ability of the network.

3D-CNN network has a variety of network structures. This paper compares C3D^42^, 3D-ResNet^38^, P3D^39^ and other networks, and tests the impact of 3D-ResNet network results at different depths on sign language recognition results. In order to verify the feasibility of the 3D-CNN network in the sign language recognition task, we compared the network recognition effect, floating-point operation amount and parameter amount after only using the global image and fusing the hand area. The results are shown in Table 4.

**Table 4:** Comparison of experimental results of different 3D-CNN networks.

Method	FLOPs (G)	Parm (M)	Top1 (%)	Top2 (%)	Top3 (%)	Top4 (%)	Top5 (%)	AVG (%)
C3D	32.998	78.405	78.34	88.44	91.70	93.72	94.80	89.40
C3D + local	98.961	145.514	87.42	94.08	96.30	97.40	98.20	94.68
3D-ResNet10	5.654	14.445	76.82	86.04	90.30	92.34	93.20	87.74
3D-ResNet10 + local	16.961	14.496	84.84	92.46	95.14	96.68	97.62	93.35
3D-ResNet34	12.696	63.548	76.58	83.62	86.24	88.10	89.26	84.76
3D-ResNet34 + local	38.088	63.599	87.90	94.04	96.14	97.30	97.84	94.64
P3D	4.192	25.073	77.50	88.74	93.32	95.10	96.08	90.15
P3D + local	12.577	25.278	72.32	83.18	87.94	90.26	92.38	85.22
**3D-ResNet18 (Baseline)**	**8.308**	**33.246**	**81.06**	**88.98**	**92.22**	**94.00**	**94.78**	**90.21**
**3D-ResNet18 + local**	**24.924**	**33.297**	**88.66**	**94.30**	**96.42**	**97.44**	**98.02**	**94.97**

Experimental results of our method are in bold.

It can be seen from Table 4 that the method of integrating hand regions can improve the accuracy of sign language recognition to a certain extent. The recognition effect of 3D-ResNet18 is the best. The accuracy of Top1 after merging the hand region reaches 88.66%, and the number of parameters and floating-point operations are 24.924 and 33.297 respectively. Although C3D network can also achieve a better recognition effect, and the Top1 accuracy after merging the hand area can reach 87.42%, due to the defects of its own structure, there are too many full connection layers and three-dimensional convolution calculations. In practical applications, the amount of parameters and floating-point operations is too large, and the cost of computing resources is too high. Therefore, this paper will not consider when selecting the network. 3D-ResNet10 is difficult to fully learn the characteristics of sign language due to the shallow layer of the network. However, 3D-ResNet34 has overfitting due to the deep depth of the network. Although data enhancement, regularization and dropout are also used to reduce overfitting, the accuracy of the test set is still lower than that of 3D-ResNet18. The P3D network decomposes the three-dimensional convolution, and its floating-point operation and parameter quantity are the least in the comparison network, but its recognition accuracy is low. After the network decomposes the convolution, it is difficult to extract sufficient space–time features from the sign language video, and the accuracy decreases after the hand region is fused. On this basis, this paper analyzes the advantages and disadvantages of several networks, and compares their accuracy, floating-point operations and parameters. On the one hand, it is necessary to ensure that the network can obtain better sign language representation. On the other hand, it is also necessary to reduce the network parameters to reduce the computing cost. Therefore, this paper selects 3D-ResNet18 as the baseline, and verifies the effectiveness of the designed residual module and the fused hand.

In order to verify the effect of ME module at different stages of 3D-ResNet18, this paper conducted ablation comparison experiments on the ME module on the basis of baseline. The experimental results are shown in Table 5. It can be seen that the ME module can effectively improve the accuracy of sign language recognition, which is more than 6% higher than the baseline method, which shows the importance of motion information for sign language recognition. Experiments show that adding the ME module to different stages of 3D-ResNet18 network can improve the recognition accuracy to a certain extent, but adding the ME module to Stage2-5 is the best. The recognition accuracy of Top1 reaches 88.80% and the average accuracy of Top1- Top5 reaches 95.94%. Compared with the baseline method, its floating-point operation and parameter amount only increased by 0.002 G and 0.06 M, but significantly improved the recognition accuracy. Therefore, in subsequent experiments, this paper added the ME module to the Stage2-5 layer of the 3D-ResNet18 network.

**Table 5:** ME module ablation test results.

Method	FLOPs (G)	Parm (M)	Top1 (%)	Top2 (%)	Top3 (%)	Top4 (%)	Top5 (%)	AVG (%)
Baseline	8.308	33.246	81.06	88.98	92.22	94.00	94.78	90.21
Baseline + ME (Stage2)	8.308	33.247	87.62	95.64	97.58	98.54	99.00	95.68
Baseline + ME (Stage3)	8.308	33.249	88.42	95.60	97.32	98.22	98.70	95.65
Baseline + ME (Stage4)	8.308	33.257	88.16	95.46	97.44	98.26	98.82	95.63
Baseline + ME (Stage5)	8.308	33.289	88.64	95.78	97.72	98.52	98.94	95.92
**Baseline + ME (Stage2–5)**	**8.310**	**33.306**	**88.80**	**95.80**	**97.72**	**98.52**	**98.86**	**95.94**

Significant values are in bold.

Through the above experiments, it can be proved that the ME module is effective for the recognition effect. Although the direct addition of the ME module does not introduce too much calculation, it does not reduce the amount of parameters in the original network. Its convolution calculation is still a three-dimensional convolution method. Therefore, the residual module designed in this paper decomposes the three-dimensional convolution into one-dimensional convolution in time and two-dimensional convolution in space. At the same time, the HSB module is introduced to enhance the extraction of spatial features. This can not only make up for the impact of decomposition on the results, but also improve the recognition accuracy of the model.

In order to verify the effect of the designed residual block and the fused hand region, the ablation contrast experiment was carried out in this paper. The experimental results are shown in Table 6. The residual module designed in this paper is added to the 3D-ResNet18 network. Different stages will have a certain impact on the recognition results. First, set the number of packets $s$ in the HSB module to 1, that is, verify the effect of 3D convolution decomposition without feature grouping. It can be seen from Table 6 that the identification result of adding residual module to Stage2 is the best. The identification accuracy of Top1 of the network is 85.70%, which is lower than that of adding the ME module only, but its parameter quantity is reduced by 0.394 M, and the floating-point operation quantity is reduced by 2.158 G. When the ME module is added to Stage4 and Stage5, it is difficult for the network to learn effective sign language representation due to the decomposition of convolution. Therefore, this paper places the improved residual block in Stage2 and carries out subsequent experiments.

**Table 6:** Experimental results of improved residual module and fused hand region ablation.

Method	FLOPs (G)	Parm (M)	Top1 (%)	Top2 (%)	Top3 (%)	Top4 (%)	Top5 (%)	AVG (%)
Baseline	8.308	33.246	81.06	88.98	92.22	94.00	94.78	90.21
Baseline + local	24.924	33.297	88.66	94.30	96.42	97.44	98.02	94.97
Baseline + ME	8.310	33.306	88.80	95.80	97.72	98.52	98.86	95.94
**Baseline + Improved residuals (s = 1, Stage2)**	**6.152**	**32.912**	**85.70**	**93.98**	**96.54**	**97.80**	**98.38**	**94.48**
Baseline + Improved residuals (s = 1, Stage3)	7.443	32.003	85.16	94.10	96.68	97.76	98.36	94.41
Baseline + Improved residuals (s = 1, Stage2, 3)	5.285	31.610	82.32	93.08	96.12	97.48	98.16	93.43
Baseline + Improved residuals (s = 2, Stage2)	6.383	32.949	86.10	93.96	96.48	97.54	98.12	94.44
Baseline + Improved residuals (s = 3, Stage2)	6.478	32.965	86.86	93.96	96.34	97.42	98.04	94.52
Baseline + Improved residuals (s = 4, Stage2)	6.517	32.971	85.12	93.20	95.60	97.00	97.60	93.70
**Baseline + Improved residuals (s = 5, Stage2)**	**6.500**	**32.968**	**88.30**	**95.54**	**97.64**	**98.42**	**98.76**	**95.73**
Baseline + Improved residuals (s = 6, Stage2)	6.490	32.967	87.62	94.88	96.64	97.68	98.14	94.99
Baseline + Improved residuals (s = 7, Stage2)	6.483	32.966	84.34	93.20	95.76	96.98	97.58	93.57
Baseline + Improved residuals (s = 8, Stage2)	6.446	32.960	85.54	93.92	96.38	97.74	98.34	94.38
**Baseline + Improved residuals (s = 5, Stage2)** ** + local(Ours)**	**19.500**	**33.019**	**91.12**	**96.34**	**97.98**	**98.68**	**98.90**	**96.60**

Significant values are in bold.

Then adjust the number of groups of HSB module to verify the impact of different feature groups on recognition accuracy. As can be seen from Table 6, when the number of groups $s = 5$, the Top1 recognition accuracy is 88.30%, which is increased by 2.6% compared with the number of groups $s = 1$, indicating that the HSB module can improve the characterization ability of features and extract richer spatial features. In this algorithm, the improved residual module is added to Stage2 of the 3D-ResNet18 network. At the same time, the number of packets of the HSB module is set to $s = 5$, and the characteristics of the hand region are fused into the recognition network. The recognition accuracy of Top1 is 91.12%, and the average recognition accuracy of Top1-Top5 is 96.60%. The floating-point operation amount and parameter amount are 19.500 and 33.019 respectively, which are reduced to a certain extent compared with the Baseline + local method.

**Figure 18:**
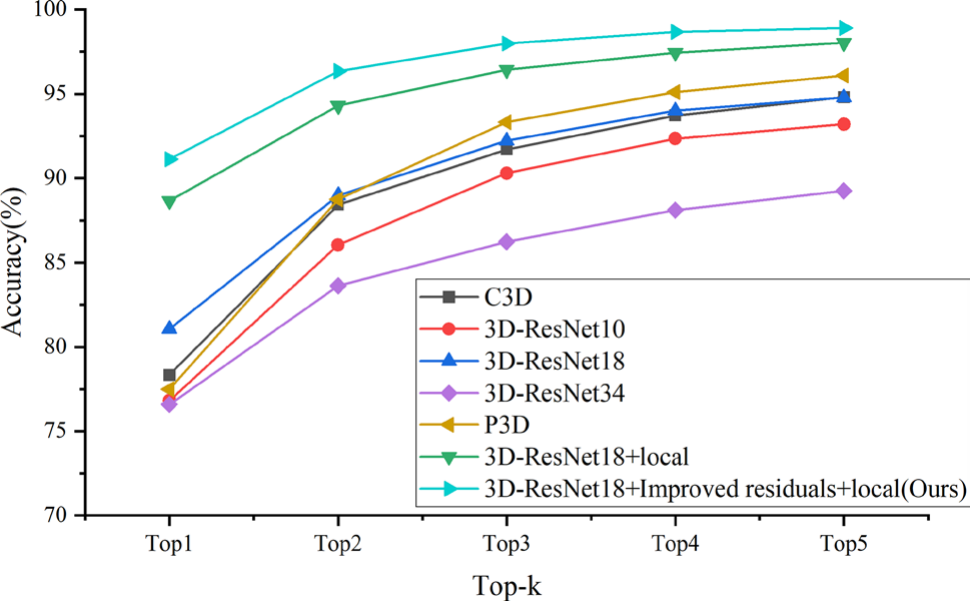
Comparison of recognition accuracy of different algorithms.

In order to more intuitively show the recognition effect of the algorithm in this paper, this paper compares the Top1–Top5 accuracy of several comparison algorithms in the form of a graph. It can be seen from Fig. 18 that the overall performance of the model in this paper is better than the comparison algorithm. After integrating the hand region and improving the residual module, the recognition accuracy has been improved to a certain extent.

The algorithm proposed in this paper has been tested and compared with several excellent algorithms^30–34^ in recent years. The results are shown in Table 7. The algorithm in the literature^30^ mainly relies on I3D and BLSTM to obtain spatiotemporal features. Reference^31^ is an algorithm based on B3D ResNet. The algorithm in document^32^ combines the global features of AM-ResC3D and the local features of RCNN. The algorithm in reference^33^ takes into account the key points and their trajectory characteristics. The algorithm in reference^34^ is a framework based on Recurrent Neural Network (RNN). The experimental results show that the recognition accuracy of the algorithm proposed in this paper is better than the comparison algorithm. According to previous experiments, this is due to the enhancement of hand features and the improvement of three-dimensional convolutional neural network, so as to extract more abundant and accurate sign language features. In addition, the local and global features of the left and right hands are fused to obtain information that is more conducive to the recognition of features, and the accuracy of sign language recognition is significantly improved.

**Table 7:** Comparative experimental results of different algorithms.

Core method/network	References	Dataset	General class	Accuracy (%)
I3D+BLSTM	Adaloglou et al.^30^	GSL isol	310	89.74
B3D ResNet	Liao et al.^31^	DEVISIGN_D	500	89.80
AM-ResC3D+RCNN	Zhang et al.^32^	DEVISIGN_D	500	91.00
Method of Fakhfakh et al	Fakhfakh et al.^33^	SIGNUM	450	90.10
Method of Xiao et al	Xiao et al.^34^	Kinect RGB-D	500	85.24 ± 1.83
Ours		CSL	500	91.12

## Conclusion

In order to fully extract the spatio-temporal features of sign language video frames, this paper proposes an improved 3D-ResNet sign language recognition algorithm to enhance hand features. On the one hand, the enhanced feature extraction module in EfficientDet hand detection network is improved, using different scale feature layers to fuse more levels of information. At the same time, the DCSAM attention module is designed to strengthen the detection ability of small targets in the network. On the other hand, the 3D-ResNet18 sign language recognition network integrates global and local feature information, improves the residual module, decomposes 3D convolution and introduces the ME module and HSB module, which not only reduces the amount of calculation, but also enhances the ability of the network to extract hand motion information and spatial information.

The algorithm proposed in this paper achieves an average accuracy of 96.6% of Top1-Top5 on the CSL datasets. Experimental results show that this algorithm has enhanced the ability of spatial feature extraction, improved the accuracy of hand detection, and still has a good detection effect in the case of hand intersection, occlusion and blur. This will be beneficial to the development and application of sign language recognition technology, and provide technical support for people with hearing or language impairment to communicate more naturally with others and better integrate into society. This technology also has reference value for human–computer interaction.

Although this algorithm is a good progress in sign language recognition, it still has some limitations. At present, the dataset available for use is small, the proposed algorithm only recognizes isolated word sign language, and the model operation speed is slow. Therefore, expanding and building sign language datasets, studying efficient and accurate continuous sentence sign language recognition algorithms, and using cloud computing^43–45^ and optimization algorithm^46–51^ to speed up the efficiency of the algorithm are important directions of follow-up research work.

## Data Availability

The data used to support the findings of this study are available from the corresponding author upon request.
